# The Predictive Value of Tumor Volume and Its Change on Short-Term Outcome for Esophageal Squamous Cell Carcinoma Treated With Radiotherapy or Chemoradiotherapy

**DOI:** 10.3389/fonc.2020.586145

**Published:** 2021-02-01

**Authors:** Shuai Liang, Chengming Li, Zhenhua Gao, Dongping Shang, Jinming Yu, Xue Meng

**Affiliations:** ^1^ Department of Radiation Oncology, Shandong Cancer Hospital and Institute, Shandong First Medical University and Shandong Academy of Medical Sciences, Jinan, China; ^2^ Department of Radiation Physics, Shandong Cancer Hospital and Institute, Shandong First Medical University and Shandong Academy of Medical Sciences, Jinan, China

**Keywords:** esophageal squamous cell carcinoma, tumor volume change, radiotherapy, gross tumor volume, short-term outcome

## Abstract

**Objectives:**

To investigate the tumor volume and its change on short-term outcome in esophageal squamous cell carcinoma (ESCC) patients who underwent definitive radiotherapy or chemoradiotherapy.

**Methods and Materials:**

All data were retrospectively collected from 418 ESCC patients who received radiotherapy or chemoradiotherapy at our institution between 2015 and 2019. Short-term outcome using the treatment response evaluation was assessed according to the RECIST 1.1. The tumor volume change rate (TVCR) was defined as follows: TVCR **=** {1 **−** [gross tumor volume (GTV) at shrinking irradiation field planning)]/(GTV at the initial treatment planning)} ×100%. Chi square test was used to compare the clinic characteristics in different TVCR groups, and the difference between initial GTV (GTVi) and shrinking GTV (GTVs) was compared using Wilcoxon’s sign rank test. Logistic regression analysis and Spearman correlation was performed.

**Results:**

There was a significant decrease in GTVi compared to GTVs (*P* < 0.001). In univariate analysis, age, cT-stage, TNM stage, treatment modality, GTVi, and TVCR were associated with short-term outcome (all *P* **<** 0.05). In multivariate analysis, gender and TVCR were statistically significant (*P* = 0.010, <0.001) with short-term outcome, and the combined predictive value of gender and TVCR exceeded that of TVCR (AUC, 0.876 *vs* 0.855).

**Conclusions:**

TVCR could serve to forecast short-term outcome of radiotherapy or chemoradiotherapy in ESCC. It was of great significance to guide the individualized treatment of ESCC.

## Introduction

Esophageal cancer (EC) has become the seventh most common tumor and the sixth leading cause of cancer death in 2018 worldwide (1). About 90% of its pathological type is esophageal squamous cell carcinoma (ESCC) in China (2). Generally speaking, surgical treatment is the best choice for early EC patients, but because of the incidence of EC is relatively not known, the early symptoms are not obvious, and it is often detected at the advanced clinical stage once patient with the obvious symptom of dysphagia. So, about 40 to 60% of the patients lose the opportunity of operation, and these patients can be only treated with radiotherapy and chemotherapy (3). Radiation sensitive patients can achieve the same survival time as surgery, especially due to the continuous improvement of the radiotherapy technology in recent years, maximize the appropriate target area, improve the target dose, and effectively protect organs at risk (OARs) and normal tissues, and significantly improve the local control rate (4).

However, because of the tumor heterogeneity, differences in tumor gene expression and tumor microenvironment, it is clinically observed that even if the EC patients have the same clinical stage, pathological type and degree of differentiation, they are also given the same radiotherapy method and dose, but the curative effect may be very different (5). Even receiving standard treatment, about 27–50% of the patients would have local recurrence and distant metastasis or both (6). Furthermore, the survival time of patients who were resistant to radiotherapy could not benefit or even be significantly shortened, and they had to bear many side effects of radiotherapy, including ulcer, esophageal fistula, pulmonary fibrosis, radiation cardiotoxicity and so on (7). Therefore, it is very necessary to predict the short-term outcome (STO) of radiotherapy in EC patients. This can help us to find potential sensitive or insensitive patients and carry out personalized and accurate treatment according to individual sensitivity in order to improve the curative effect, the local control rate and prolong the survival time.

The gross tumor volume (GTV) defined on radiotherapy planning refers to the range of tumor lesions with certain shape and size displayed by existing auxiliary examination methods including computed tomography (CT) and esophagography, and its stereoscopic imaging reflects the actual shape of the tumor. With the improvement of radiotherapy technology, we have ability to routinely evaluate GTV during radiotherapy, and it has been confirmed as a predictor in non-small cell lung cancer (8, 9) and nasopharyngeal carcinoma (10, 11). Although GTV as a prognostic factor has been studied in EC treated by surgery alone, no studies investigated the tumor volume change rate (TVCR) during radiotherapy (12, 13). In clinical work, we found that STO after treatment was the important guiding significance for indicating tumor recurrence, metastasis. So, in this study, we conducted GTV and TVCR in ESCC patients during radiotherapy and investigate whether it is a predictor of STO with the aim of making individualized treatment as soon as possible.

## Methods and Materials

### Characteristics of Patients

This retrospective study included 418 cases of ESCC patients who received radiotherapy or chemoradiotherapy (CRT) in Shandong Cancer Hospital between October 2015 and May 2019. Inclusion criteria include the following: (1) patients were aged 18 years or older; (2) new diagnosed ESCC and confirmed by histopathology; (3) receipt of ≥50Gy radiotherapy with or without chemotherapy, and a repeated computed tomography (CT) scan was performed before and follow 40Gy for shrinking irradiation field; (4) availability of dosimetry, radiotherapy planning evaluation system and imaging data; (5) the local focus could not be resected; (6) Karnofsky performance status ≥70; (7) no past history of malignant tumors, no intolerable serious medical diseases. The exclusion criteria included: (1) distant metastasis; (2) esophagography showed signs of esophageal perforation; (3) active esophageal bleeding; (4) complete esophageal obstruction. This was a retrospective study, and it was approved by the Ethics Committee of Shandong Cancer Hospital, and informed consents were obtained from all included individuals.

### Radiotherapy

The GTV was identified by diagnostic and radiotherapy planning CT images and esophagography. All patients were treated with 3-dimensional conformal radiotherapy or intensity-modulated radiotherapy. Radiotherapy alone, radical simultaneous or sequential CRT was performed according to the clinical stage. The delineation of target volumes and OARs referred to the Radiotherapy and Oncology Group (RTOG) guidelines. All radiotherapy plans were generated in the Eclipse system (Varian Medical Systems, Palo Alto, CA, Version 13.5.35), and delivered with 6 MV photons beams. The prescribed doses of radiotherapy were 45–66 Gy at 1.8–2.0 Gy per fraction once daily and five fractions per week. Plans were normalized to 95% of the plan tumor volume received 100% of the prescribed dose. The dose of all OARs was controlled below the safe range.

### Chemotherapy

Some patients underwent concurrent or sequential CRT based on individualized treatment strategy. The regimens mainly consisted of two kinds of platinum-based chemotherapy. One was cisplatin with fluorouracil, one was cisplatin with paclitaxel. The doses of chemotherapy regimens followed the guidelines of Chinese Society of Clinical Oncology and National Comprehensive Cancer Network for EC.

### Response Evaluation

All patients performed esophagography and enhanced CT of chest, abdomen and neck before and at 1 month after radiotherapy or CRT. The imaging data were analyzed and the STO of all patients were assessed by an imaging deputy chief physician and a radiotherapy deputy chief physician according to Response Evaluation Criteria in Solid Tumor 1.1 (RECIST 1.1) without knowledge of the results of TVCR studies. Patients with an outcome of complete response (CR) or partial response (PR) were subsequently classified as responders according to RECIST 1.1. Meanwhile, those who had an outcome of stable disease (SD) or progressive disease (PD) were defined as non-responders.

### TVCR Calculation

We defined GTV including the primary tumor and involved lymph nodes at initial treatment planning as GTVi, at shrinking irradiation field planning as GTVs. All GTVi and GTVs were extracted from the Varian treatment planning system. The TVCR were calculated as follows:

TVCR=(1−GTVs/GTVi) × 100%

### Statistical Analysis

Using receiver operating characteristic (ROC) curve to convert continuous variables into binary variables. Chi square test was used to compare the clinic characteristics in different TVCR, and the difference between GTVi and GTVs was compared using Wilcoxon’s sign rank test. Univariate logistic regressions analysis was performed to estimate the odds ratio (OR) and confidence interval (CI) to evaluate the effect of independent variables on STO. In order to avoid omitting indicators that might be of clinical significance, factors that had *P <*0.1 in univariate analyses were subjected to multivariate analysis. Spearman’s rank correlation coefficient (*r*) values were calculated to analyze how other independent variables related to TVCR. A significant difference was considered if a *P* value was <0.05 in two-sided. And all statistical analyses were conducted with IBM SPSS Statistics 25.0 software (SPSS Inc., Chicago, IL).

## Results

### Clinical Parameters of the Patients

**Figure 1:**
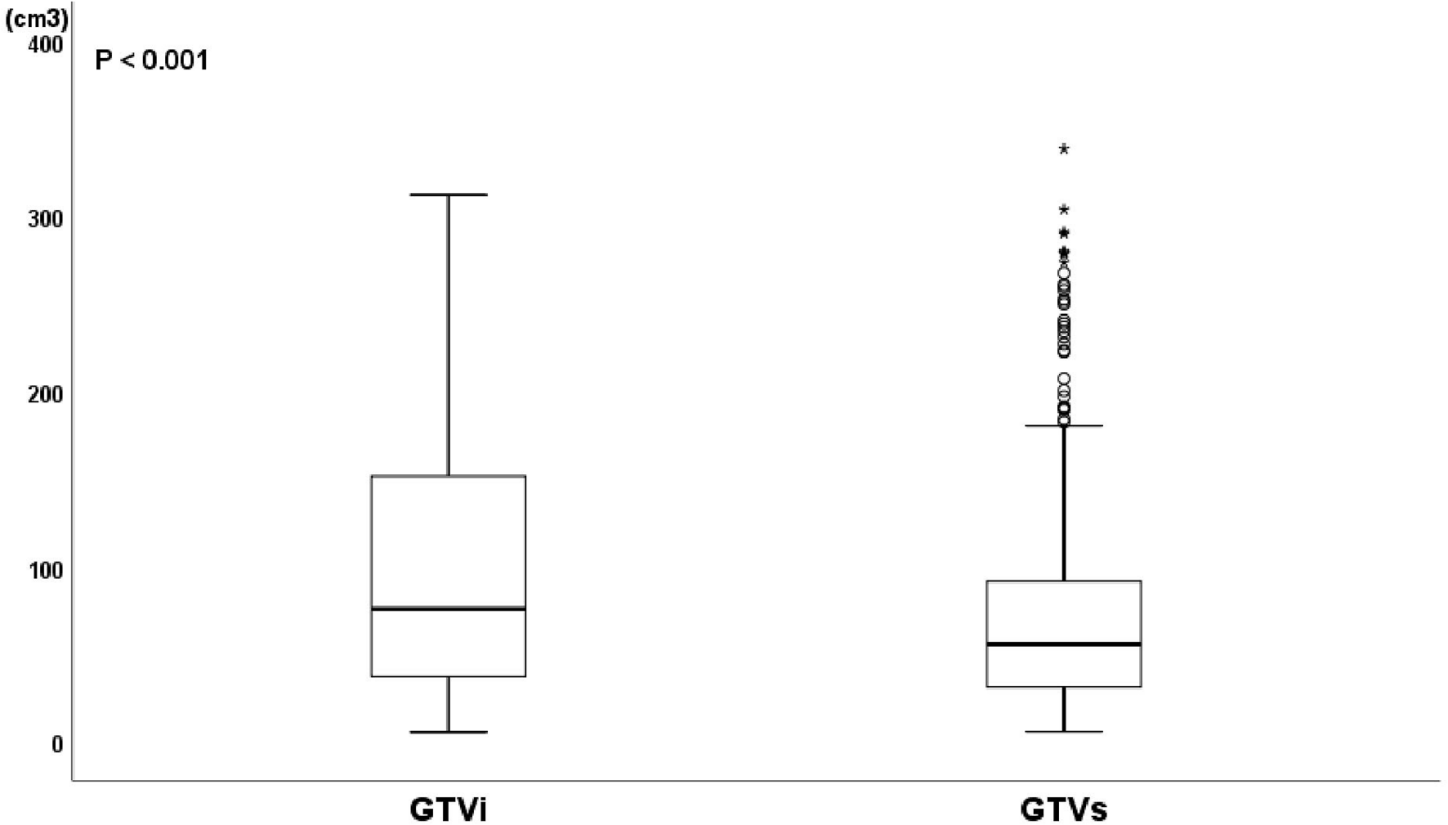
Boxplot of GTV at initial treatment planning (GTVi) and GTV at shrinking irradiation field planning (GTVs). Wilcoxon’s signed rank test, P < 0.001.

**Figure 2:**
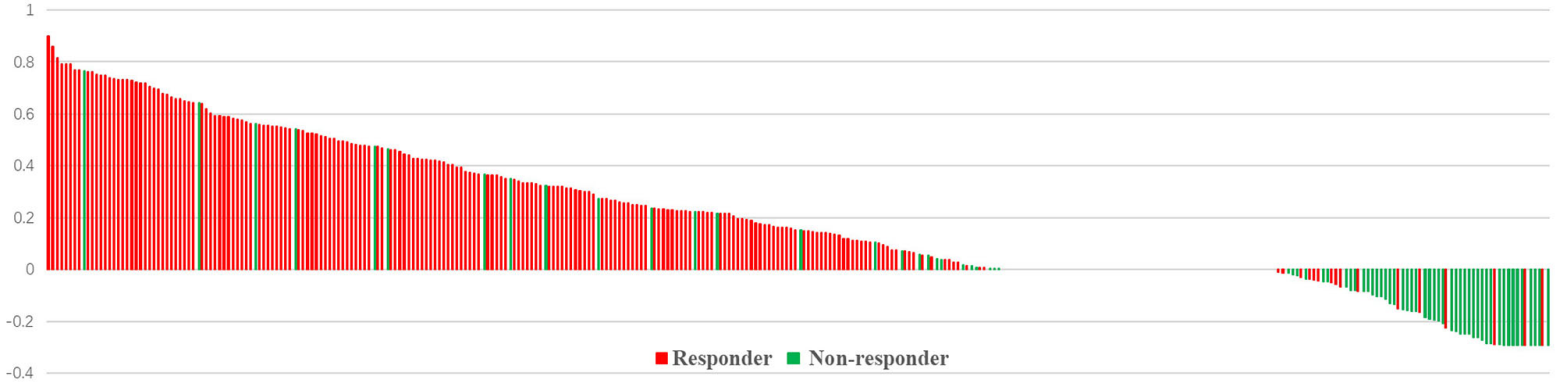
Individual changes in tumor volume at shrinking irradiation field planning. Bar length indicates the tumor volume change rate. Patients with responder (red) or non-responder (green).

The median age and radiation dose were 70 years (range, 45–92 years) and 60 Gy (range, 50–66 Gy), respectively. The optimal cut-off values of GTVi, GTVs, and TVCR were 59.45 (range, 5.4–312.0 cm^3^; sensitivity 51.4%; specificity 51.4%; 95%CI, 0.526–0.644), 55.78 (range, 5.7–338.0 cm^3^; sensitivity 64.7%; specificity 54.1%; 95%CI, 0.526–0.644) and 6.155% (range, −29.3 to 90.0%; sensitivity 68.8%; specificity 92.5%; 95%CI, 0.819–0.891), respectively. 227 patients (54.3%) underwent concurrent or sequential CRT, and 191 patients (45.7%) only received radiotherapy. All patient characteristics were listed in **Table 1**. The tumor volume changes in all patients at shrinking irradiation field planning were as follows: 217 patients (51.9%) had a decrease (mean ± standard deviation, 50.91 ± 56.82); 139 patients (33.3%) showed no change; and 62 patients (14.8%) demonstrated an increase (mean ± standard deviation, 6.635 ± 7.116). There was a significant decrease in GTVi (mean ± standard deviation, 100.5 ± 79.54) compared to GTVs (mean ± standard deviation, 75.03 ± 63.52), with GTVi decreasing by a median of 1.67% (range **−**29.3 to 90.4%; *P*<0.001) (**Figure 1**). At 1 month after radiotherapy or CRT, 272 patients (65.1%) were assessable for responders, 146 patients (34.9%) were assessable for non-responders. And individual change rate in tumor volume at shrinking irradiation field planning were shown in **Figure 2**. According to cut-off values, we also divided all patients into high TVCR group (≥6.155%) and low TVCR group (<6.155%). There was significant correlation between the GTVi with high and low TVCR groups (χ^2^ = 24.435, *P* < 0.001), which was shown in the **Table 2**.

**Table 1:** Baseline characteristics of all patients.

Characteristics	Total	Responder	Non-responder
	**No (%) N = 418**	**N = 272 (%)**	**N = 146 (%)**
Age (years)			
<70	211 (50.5)	148 (54.4)	63 (43.2)
≥70	207 (49.5)	124 (45.6)	83 (56.8)
Gender			
Male	296 (70.8)	185 (68.0)	111 (76.0)
Female	122 (29.2)	87 (32.0)	35 (24.0)
Location			
Cervical	54 (12.9)	37 (13.6)	17 (11.6)
Thoracic			
Upper	119 (28.5)	80 (29.4)	39 (26.7)
Middle	113 (27.0)	71 (26.1)	42 (28.8)
Lower	132 (31.6)	84 (30.9)	48 (32.9)
cT-stage			
T2	66 (15.8)	35 (12.9)	31 (21.2)
T3	279 (66.7)	187 (68.7)	92 (63.0)
T4	73 (17.5)	50 (18.4)	23 (15.8)
cN-stage			
N0	115 (27.5)	68 (25.0)	47 (32.2)
N+			
N1	203 (48.6)	136 (50.0)	67 (45.9)
N2	87 (20.8)	60 (22.1)	27 (18.5)
N3	13 (3.1)	8 (2.9)	5 (3.4)
TNM stage			
IIA/IIB	133 (31.8)	76 (27.9)	57 (39.0)
IIIA/IIIB	202 (48.3)	139 (51.1)	63 (43.2)
IVA	83 (19.9)	57 (21.0)	26 (17.8)
Treatment modality			
No chemotherapy	191 (45.7)	114 (41.9)	77 (52.7)
Chemoradiotherapy			
Concurrent	172 (41.1)	121 (44.5)	51 (34.9)
Sequential	55 (13.2)	37 (13.6)	18 (12.3)
Chemotherapy			
No	191 (45.7)	114 (41.9)	77 (52.7)
PF regimen	126 (30.1)	86 (31.6)	40 (27.4)
TP regimen	101 (24.2)	72 (26.5)	29 (19.9)
GTVi (cm^3^)			
<59.45	175 (41.9)	96 (35.3)	79 (54.1)
≥59.45	243 (58.1)	176 (64.7)	67 (45.9)
GTVs (cm^3^)			
<55.78	211 (50.5)	140 (51.5)	71 (48.6)
≥55.78	207 (49.5)	132 (48.5)	75 (51.4)
Radiation dose (Gy)			
<60	200 (47.8)	130 (47.8)	70 (47.9)
≥60	218 (52.2)	142 (52.2)	76 (52.1)
TVCR (%)			
<6.155	220 (52.6)	85 (31.25)	135 (92.5)
≥6.155	198 (47.4)	187 (68.75)	11 (7.5)

TVCR, tumor volume change rate; PF regimen, cisplatin + fluorouracil; TP regimen, paclitaxel + cisplatin; GTVi, GTV at initial treatment planning; GTVs, GTV at shrinking irradiation field planning; clinical T stage, N stage, TNM stage, Clinical cancer stage according to the American Joint Committee of Cancer eighth edition TNM classification and staging system.

**Table 2:** Comparison of tumor volume change rate (TVCR) and clinical characteristics.

Characteristics	High TVCR group	Low TVCR group	χ^2^	*P*
	No (%). N = 220	No (%). N = 198		
Age (years)			3.146	0.076
<70	102 (46.4)	109 (55.1)		
≥70	118 (53.6)	89 (44.9)		
Gender			0.149	0.700
Male	154 (70.0)	142 (71.7)		
Female	66 (30.0)	56 (28.3)		
Location			5.772	0.123
Cervical	32 (14.5)	22 (11.1)		
Thoracic				
Upper	52 (23.6)	67 (33.8)		
Middle	61 (27.7)	52 (26.3)		
Lower	75 (34.1)	57(28.8)		
cT-stage				
T2	41 (18.6)	25 (12.6)	4.650	0.098
T3	147 (66.8)	132 (66.7)		
T4	32 (14.5)	41 (20.7)		
cN-stage			1.142	0.767
N0	64 (29.1)	51 (27.5)		
N1	104 (47.3)	99 (50.0)		
N2	44 (20.0)	43 (21.7)		
N3	8 (3.6)	5 (2.5)		
TNM stage			2.325	0.313
IIA/IIB	75 (34.1)	58 (29.3)		
IIIA/IIIB	107 (48.6)	95 (48.3)		
IVA	38 (17.3)	45 (22.7)		
Treatment modality			0.467	0.495
Radiotherapy	104 (47.3)	87 (43.9)		
Chemoradiotherapy	116 (52.7)	111 (56.1)		
GTVi (cm^3^)			24.435	<0.001
<59.45	117 (53.2)	58 (29.3)		
≥59.45	103 (46.8)	140 (70.7)		
GTVs (cm^3^)			0.630	0.427
<59.45	107 (48.6)	104 (52.5)		
≥59.45	113 (51.4)	94 (47.5)		

TVCR, tumor volume change rate; GTVi, GTV at initial treatment planning; GTVs, GTV at shrinking irradiation field planning; clinical T stage, N stage, TNM stage, Clinical cancer stage according to the American Joint Committee of Cancer eighth edition TNM classification and staging system.

### Univariate and Multivariate Analyses for Short-Term Outcome

**Figure 3:**
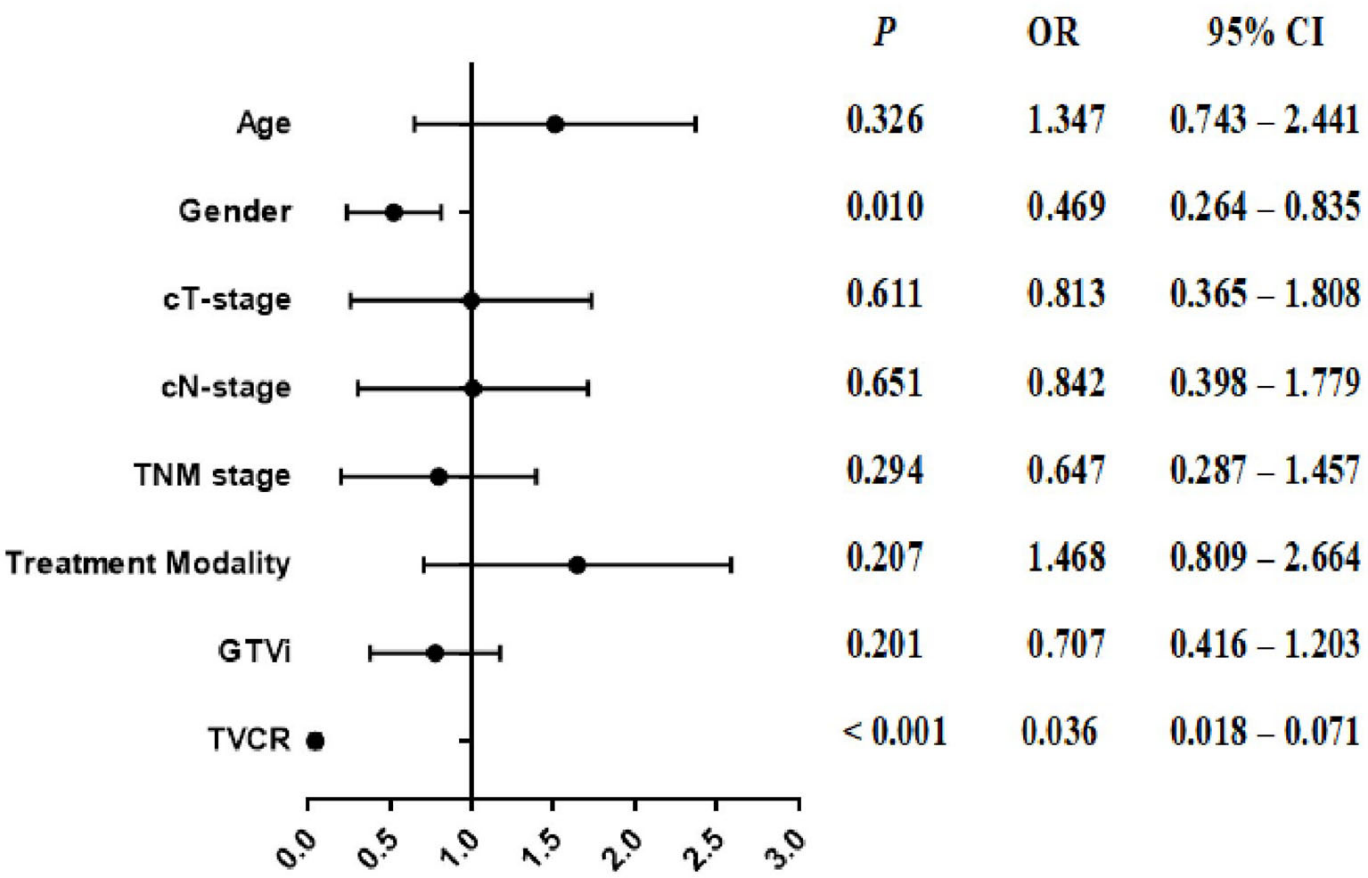
The forest plot of the multivariate analysis of clinical, and tumor volume in predicting the short-term outcome.

**Figure 4:**
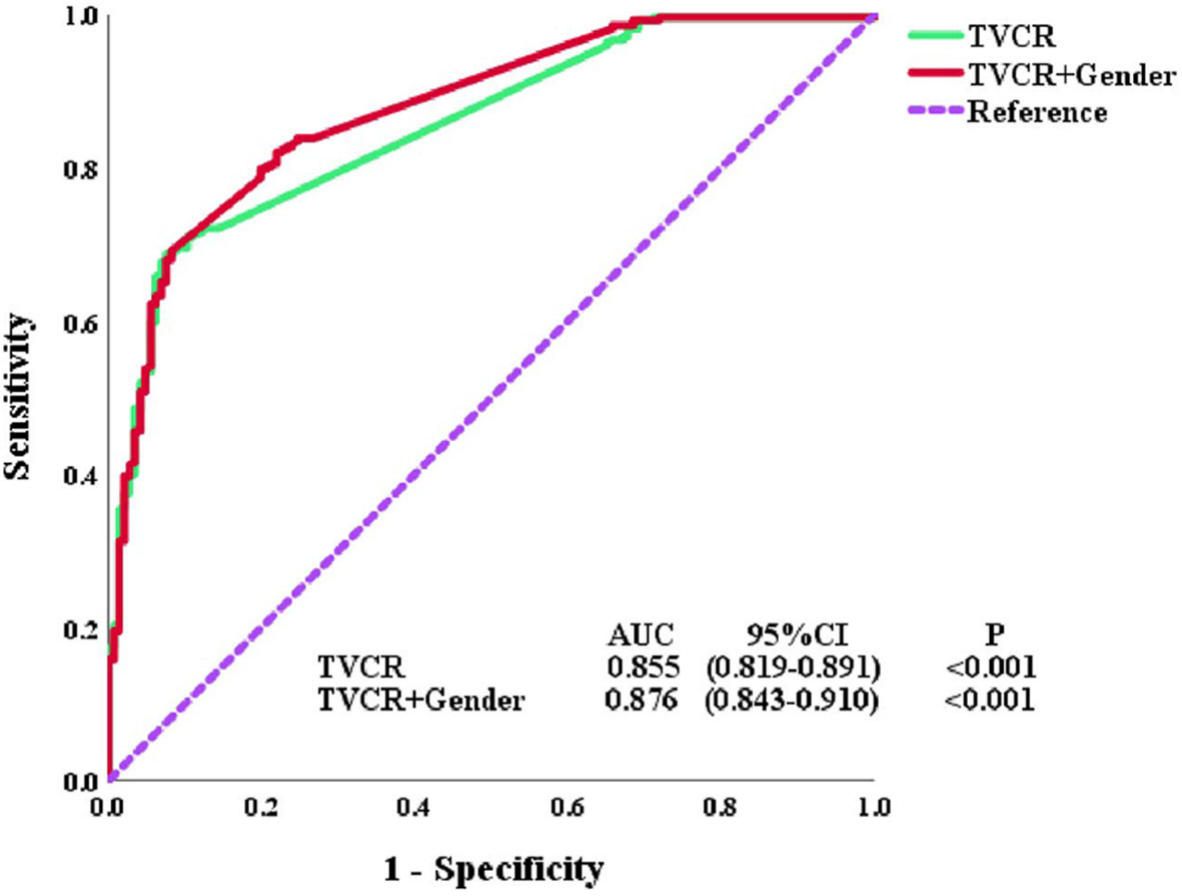
Receiver operator characteristic (ROC) curves for tumor volume change rate (TVCR) and multivariate.

In univariate analysis, age [OR, 1.572; 95% CI, 1.049–2.358; *P* = 0.029], cT-stage [OR, 0.548; 95% CI, 0.322–0.933; *P* = 0.027], TNM stage [OR, 0.576; 95% CI, 0.381–0.870; *P* = 0.009], treatment modality [OR, 1.547; 95% CI, 1.032–2.318; *P* = 0.035], GTVi [OR, 0.463; 95% CI, 0.307–0.697; *P* < 0.001], and TVCR [OR, 0.037; 95% CI, 0.019–0.072; *P* < 0.001] were significantly associated with STO (**Table 3**). The variables with *P <*0.1 in univariate analysis were subjected to multivariate analysis. In addition to TVCR [OR, 0.036; 95% CI, 0.018–0.071; *P* < 0.001], gender [OR, 0.469; 95% CI, 0.264–0.835; *P* = 0.010] was also a potential factor which could predict STO (**Figure 3**). And the combined predictive value (AUC, 0.876; 95%CI, 0.843–0.910; P < 0.001) of gender and TVCR is exceeded of TVCR (AUC, 0.855; 95%CI, 0.819–0.891; P < 0.001) (**Figure 4**).

**Table 3:** Univariate logistic analysis for short-term outcome.

Characteristics	*P* value	OR	95% CI
Age (<70 *vs*. ≥70), years	0.029	1.572	1.049–2.358
Gender (Male *vs*. Female)	0.087	0.670	0.424–1.059
Location (Cervical *vs*. Thoracic)	0.569	1.195	0.647–2.206
cT-stage (T2 *vs*. T3 & T4)	0.027	0.548	0.322–0.933
cN-stage (N0 *vs*. N+)	0.064	0.681	0.454–1.022
TNM stage (II *vs*. III & IVA)	0.009	0.576	0.381–0.870
Treatment modality (RT *vs*. CRT)	0.035	1.547	1.032–2.318
Radiation dose (<60 *vs*. ≥60), Gy	0.976	0.994	0.665–1.487
GTVi (<59.45 *vs*. ≥59.45), cm^3^	<0.001	0.463	0.307–0.697
GTVs (<55.78 *vs*. ≥55.78), cm^3^	0.580	1.120	0.665–1.487
TVCR (<6.155 *vs*. ≥6.155%)	<0.001	0.037	0.019–0.072

RT, Radiotherapy; CRT, Chemoradiotherapy; GTVi, GTV at initial treatment planning; GTVs, GTV at shrinking irradiation field planning; TVCR, tumor volume change rate; clinical T stage, N stage, TNM stage, Clinical cancer stage according to the American Joint Committee of Cancer eighth edition TNM classification and staging system.

### Spearman Correlation Coefficient in Tumor Volume Change Rate

Further correlation studies indicated that TVCR was positively correlated with GTVi (*r* = 0.413, *P* < 0.001). The *P* value of TVCR with age, location, cT-stage, and TNM stage were all <0.05, but *r* value of them were <0.2 (**Table 4**).

**Table 4:** Correlation analysis between clinical factors and tumor volume change rate (TVCR).

Correlation analysis	Correlation coefficient (*r*)	*P* value
TVCR and age	−0.169	0.001
TVCR and gender	−0.014	0.774
TVCR and location	−0.150	0.002
TVCR and cT-stage	0.126	0.010
TVCR and cN-stage	0.076	0.123
TVCR and TNM stage	0.115	0.019
TVCR and treatment modality	−0.076	0.122
TVCR and primary GTVi	0.413	<0.001

### Subgroup Analysis

Gender [OR, 0.287; 95% CI, 0.107–0.768; *P* = 0.013] and TVCR [OR, 0.042; 95% CI, 0.015–0.116; *P* < 0.001] were independent predictors of STO in multivariate analysis for patients with big GTVi (≥59.45 cm^3^) (**Table 5**).

**Table 5:** Univariate and multivariate logistic regressions analysis for short-term outcome (STO) in big GTVi group (≥59.45 cm^3^).

Characteristics	Univariate analysis		Multivariate analysis	
	OR (95% CI)	*P* value	OR (95% CI)	*P* value
Age (<70 *vs*. ≥70), years	2.470	0.002	2.025	0.137
	(1.380–4.421)		(0.799–5.133)	
Gender (Male *vs*. Female)	0.414	0.026	0.287	0.013
	(0.190–0.900)		(0.107–0.768)	
Location (Cervical *vs*. Thoracic)	1.377	0.511		
	(0.530–3.577)			
cT-stage (T2 *vs*. T3 & T4)	0.621	0.227		
	(0.287–1.345)			
cN-stage (N0 *vs*. N+)	0.558	0.052	0.911	0.820
	(0.309–1.005)		(0.411–2.021)	
TNM stage (II *vs*. III & IVA)	0.768	0.386		
	(0.422–1.395)			
Treatment modality				
(Radiotherapy *vs*. Chemoradiotherapy)	2.505	0.002	1.594	0.319
	(1.406–4.462)		(0.637–3.986)	
GTVs, (<55.78 *vs*. ≥55.78), cm^3^	1.015	<0.001	1.005	0.081
	(1.010–1.020)		(0.637–3.986)	
Radiation dose (<60 *vs*. ≥60), Gy	0.842	0.549		
	(0.479–1.479)			
TVCR (<6.155 *vs*. ≥ 6.155%)	0.031	<0.001	0.042	<0.001
	(0.012–0.076)		(0.015–0.116)	

## Discussion

At present, the standard treatment scheme recommended by the guidelines for inoperable patients with locally advanced EC is concurrent or sequential CRT. But it is not clear whether further consolidation therapy is needed after CRT. How to select patients who need further consolidation therapy after CRT has become an urgent problem to be solved in clinical work. Furthermore, the main evaluation criteria of RECIST 1.1 is to measure changes in the longest diameter of assessable lesions, while ignoring short diameter and tumor volume. So, we assessed the relationship with GTVi, TVCR and STO, demonstrating that TVCR was a strong prognostic factor for unresectable ESCC patients who underwent radiotherapy or CRT. Moreover, patients with TVCR ≥6.155% during radiotherapy or CRT could have better STO. This was the first time to report the significance of TVCR during radiotherapy or CRT for ESCC. And TVCR would be a feasible predictive factor to find patients who are not sensitive to radiotherapy and develop individualized treatment as soon as possible.

GTV changes during radiotherapy or CRT differ widely in different studies. In our study, there were 62 patients (14.8%) whose GTVs were bigger than GTVi. Similar to our study, Wang et al. have reported that some patients (four of 11, 36.4%) with EC demonstrated an increase in GTV at week 2 during radiotherapy, and there were still some patients (three of 11, 27.3%) demonstrated an increase in GTV at week 4 (14). Christina et al. have also found that some patients (three3 of 19, 15.8%) with high grade glioma demonstrated an increase in GTV at week 3 during radiotherapy (15). One reason was the surrounding of the tumor might be edema after radiotherapy that made it difficult to identify whether it is the tumor invasion or the acute radiation reaction of the normal esophageal mucosa during radiotherapy, or be further enlarged without obvious retraction at the initial stage of radiotherapy. Barker et al. have also reported that tumor growth would accelerate at the initial stage of radiotherapy (16). Another reason might be the error by CT image parameters, which was compared with enhanced CT for localization, the image of tumor tissue on plain scan CT, was similar to that of surrounding normal tissue, which lead to the increase of GTV.

GTVi as a predictive factor has been confirmed in many studies. Bradley et al. reported that GTV had a high prognostic value in patients with unresectable non-small cell lung cancer (8). Chua et al. also reported that larger GTV are also associated with inferior local control rates in malignancies of the nasopharynx, larynx, and hypopharynx (10). In general, larger GTV means a heavier tumor load, more radiation-resistant hypoxic tumor cells and clonal cells, and greater restrictions on related organs that could lead to poor survival. Meanwhile, our study showed that GTVi was significant with STO in univariate analyses. Similar to our results, Chen et al. found that GTV could serve as a good prognostic factor for ESCC patients underwent radiotherapy, and Créhange et al. reported that tumor volume affected outcomes of EC (17, 18). But it was not an independent factor in our study. This might be that 54.3% of the patients in our study received CRT. So, the predictive value of GTVi still needs to be further explored.

Tumor is constantly changing during treatment and it seems that the rate of change between GTVs and GTVi which we defined as TVCR is more reasonable than GTVi in predicting effect. From disease regression, one potential advantage of quantifying TVCR during radiotherapy was to redefine the tumor volume to assess curative effect. Previous studies in other primary sites found that the larger reduction in tumor volume resulted in better local control, better disease-free survival, and better OS in cervical, non-small-cell lung and head-and-neck cancers, but some studies believed that tumor regression rate was not a predictor of survival (19–22). In our study, TVCR was a powerful predictor for STO. Similar with our result, Yang et al. have found TVCR during radiotherapy could be used as an independent factor affecting survival rate in head-and-neck cancer (21). As expected, our data demonstrated a significant correlation of GTVi with tumor volume (*r* = 0.413). However, GTVi was not independent predictors of STO in our study, suggesting that TVCR may be a more sensitive indicator. The TVCR showed a better predictive value (AUC, 0.855; 95% CI, 0.819–0.891; *P* < 0.001) on STO in this study. All GTVi and GTVs parameters came from the evaluation system after the target area was sketched. But, at present, the target and OARs are still sketched manually by doctors in clinical radiotherapy, while many factors such as doctors’ clinical knowledge, experience, energy and status determine that there are great differences in drawing quality between different doctors and different patients (23). With the development of artificial intelligence (AI) in the field of precision radiotherapy, it is possible to solve these problems. Pinnacle et al. initially realized the automatic delineation of regions of interest using atlas template library; Google developed a set of AI target delineation system based on atlas, which automatically delineated head and neck tumor lesions through machine learning (24); Sims et al. used the atlas tool to automatically draw the brainstem, parotid gland, and mandible of patients, and compared the results with the results of manual sketching (25); Lin et al. used AI technology to automatically draw nasopharyngeal tumors on magnetic resonance images, which provided a solution for accurate and efficient delineation of radiotherapy targets for nasopharyngeal carcinoma (26). All these show that AI plus radiotherapy can improve the accuracy radiotherapy and promote the automation and intelligence of radiotherapy.

Many prognostic factors achieved significance in our study, but TVCR still maintained significance for STO in multivariate analysis. Meanwhile, gender was also significant for STO in multivariate analysis. Similar with our results, Pierre et al. have found that gender is an independent prognostic factor for patients with ESCC, and female gender was a positive prognostic factor (27). In addition, studies have shown that tumor location was also related to prognosis, but in this study, tumor location was very weakly negative correlated with TVCR (*r* = −0.150). This was might be that thoracic EC spread unnoticed before the appearance of the first symptom. Créhange et al. have reported that tumor volume was correlated with tumor location, and tumors below the carina had a worse prognosis (18). They all indicated that the individualized treatment was becoming more and more important. And the combined predictive value of gender and TVCR exceeded that of TVCR (AUC 0.876 *vs* 0.855).

Several limitations would be addressed here. First, this was a retrospective, single-center study that was inevitably affected by a number of confounding factors. Second, although esophagography, CT and other auxiliary examinations were used, compared with pathological TNM staging, clinical TNM staging was still not accurate. Last, the combined of gender and TVCR had a good predictive value, but it was not tested in clinic, and its clinical application value remained to be determined.

In conclusion, our study confirmed that STO could be predicted by the changes of GTV before and during radiotherapy or chemoradiotherapy for ESCC, which had an important clinical significance for adjusting the treatment strategy and guiding individualized treatment.

## Data Availability Statement

The datasets generated for this study are available on request to the corresponding author.

## Ethics Statement

The studies involving human participants were reviewed and approved by The Ethics Committee of Shandong Cancer Hospital. The patients/participants provided their written informed consent to participate in this study. Written informed consent was obtained from the individual(s) for the publication of any potentially identifiable images or data included in this article.

## Author Contributions

XM and JY contributed to the conception and design of the study. SL and CL organized the database. SL, ZG, and DS performed the statistical analysis. SL and XM wrote the first draft of the manuscript. All authors contributed to the article and approved the submitted version.

## Funding

This work was supported by the National Natural Science Foundation of China (81972864), Academic Promotion Program of Shandong First Medical University (2019RC002), Science and Technology Support Plan for Youth Innovation Teams of Universities in Shandong Province (2019KJL001) and Science and Technology Plan of Jinan (201907113).

## Conflict of Interest

The authors declare that the research was conducted in the absence of any commercial or financial relationships that could be construed as a potential conflict of interest.
